# Nursing Student Perspectives and Curricular Insights on Health Equity Content: Findings From a Mixed Methods Study

**DOI:** 10.1111/jan.70325

**Published:** 2025-10-23

**Authors:** Anna Graefe, Lucy Mkandawire‐Valhmu, Linda Bane Frizzell, Carolyn M. Porta

**Affiliations:** ^1^ School of Nursing University of Minnesota Minneapolis Minnesota USA; ^2^ St. Olaf College Northfield Minnesota USA; ^3^ School of Public Health University of Minnesota Minneapolis Minnesota USA

**Keywords:** consciousness, health equity, nursing education, nursing education research

## Abstract

**Aims:**

(1) Explore prelicensure baccalaureate nursing students' perceptions about applying any health equity knowledge gained in school for their future nursing practice. (2) Evaluate whether student perspectives of health equity learning in their programs align with explicit health equity content within curricular documents.

**Design:**

Mixed methods explanatory sequential design.

**Methods:**

Curricular documents from nursing programs were analysed for health equity content and quantitised. Nursing students then participated in focus group interviews. Findings were analysed separately then integrated and interpreted.

**Results:**

Three nursing programs participated, including 197 curricular documents from 60 courses. Six students participated in focus group interviews. Most nursing courses contained at least one occurrence of explicit health equity content, but certain topic areas were lacking or absent. Focus group interview themes included the value of experiential learning, the process of learning through the unknown and student conscientisation but not yet fully engaging in praxis. Integration provided contextualisation of results and revealed areas of convergence and divergence.

**Conclusion:**

This mixed method study highlighted areas of alignment and conflict between student perspectives of their learning and explicit content in curricular documents. The study underscored students' perceived value of experiential learning and identified potential content gaps, including historic context and policy, aligning with calls for nurse activism.

**Implications:**

To make strides toward health equity in practice, additional focus on health equity‐related competencies in nursing education and educational research may be warranted.

**Impact:**

Nurses, both globally and in the United States, due to the size of the workforce and their unique role, are positioned to progress communities toward health equity. Nursing education is an important systemic aspect of equipping the nursing workforce to meet complex health and wellness and societal needs.

**Reporting Method:**

Journal Article Reporting Standards–Mixed Methods Article Reporting Standards guidelines.

**Patient or Public Contribution:**

No patient or public involvement.

**Summary**

- What does this paper contribute to the wider global clinical community?
  ○ Insight into nursing students' perspectives including their value of experiential learning and wish for more application of health equity‐related knowledge and skills as they prepare to enter clinical practice.
  ○ Integrated findings highlight potential differences in data sources for nurse educators to consider when incorporating and evaluating local and global health equity content in their curricula.

## 1 Introduction and Background

Between and within countries, whether low‐, middle‐ or high‐income, there are vast inequities in health outcomes between social groups (World Health Organization 2018). The systematic differences in health outcomes between groups of people arise from unevenly distributed social conditions and are called health inequities (World Health Organization 2018). Health equity is ‘the fair and just opportunity for all people to achieve their full health potential’ with no one disadvantaged due to ‘personal characteristics, historical oppression and societal influences’ (Lewis et al. 2023, 8). In the United States (US), where despite spending more on healthcare than any other country, progress toward better health for all people has been limited (Gunja et al. 2023). The *Ending Unequal Treatment* report was recently released by the National Academies of Sciences, Engineering, and Medicine [NASEM] (2024), over 20 years after an initial landmark report (Smedley et al. 2003) that documented health inequities rooted in historic and current socioeconomic inequalities. *Ending Unequal Treatment* highlighted that despite some progress in addressing health inequities in the US, ‘movement has been slow, uneven, inconsistent, and, at times, regressive among certain populations and across disease conditions’ (NASEM 2024, 308). Specific examples of health inequities across dimensions, many of which intersect, including race, ethnicity, socioeconomic position, gender identity, sexual orientation, disability and geographic location, are well established in the health care literature (NASEM 2024; World Health Organization 2018). See Table 1 for further definitions of equity‐related terms.

**TABLE 1:** Health equity‐related terms and definitions.

Term	Definition	Definition source
Health inequity	Unjust ‘systematic differences in the health status of different population groups’	World Health Organization (2018)
Health equity	‘The fair and just opportunity for all people to achieve their full health potential’ and no one is disadvantaged due to ‘personal characteristics, historical oppression and societal influences’	Lewis et al. (2023, 8)
Social justice	‘A form of justice that engages in social criticism and social change. Its focus is the analysis, critique, and change of social structures, policies, laws, customs, power, and privilege. It examines the way in which these disadvantage or harm vulnerable social groups through marginalisation, exclusion, exploitation and voicelessness’	American Nurses Association (2025) glossary
Critical consciousness	The ongoing development of a deeper awareness of the social realities of the world, including privilege and oppression, and one's ability to change that reality through critical reflection and action	Freire (1970) and Jemal (2017)
Conscientisation	‘The process that uses such tools as critical dialogue, reflective questions, and social action projects, to develop critical consciousness’	Jemal (2017, 16)
Praxis	A cyclical process of reflection and action, simultaneously or in sequence, toward transformation	Freire (1970) and Jemal (2017)

*Note:* Jemal (2017) described that the concepts of critical consciousness, conscientisation and praxis are overlapping and operate in tandem.

Despite these challenges, there is a huge potential for nurses to make a difference. As the largest sector of the healthcare workforce with over 29 million nurses estimated globally (Mathieu et al. 2022) and over five million registered nurses in the US alone (Smiley et al. 2023), nurses are uniquely equipped to meet societal needs. Nursing has a long history of population and public health efforts rooted in social justice (Darcy‐Mahoney et al. 2020; Rudner 2021). At the same time, health equity topics in nursing often remain at the periphery, with the dominance of whiteness, racism, ableism and care of the individual permeating nursing education and practice (Bonini and Matias 2021; Darcy‐Mahoney et al. 2020; Graefe et al. 2024; Iheduru‐Anderson and Waite 2022; Thurman et al. 2019). Current calls within nursing to lead equity efforts (NASEM 2021) are in line with the longstanding mission of the profession and support a needed shift in the discipline for nurses to engage in meaningful change.

Nursing education is an important and systemic aspect of preparing the nursing workforce to be equipped to meet the global and national health and societal needs. The presence of explicit health equity‐related content in nursing education or resulting student learning and competence is not well understood (Graefe et al. 2024). Particularly, research on health equity content in nursing related to cultural and social values, governance and policy and history and historic context is lacking (Graefe et al. 2024). Nurse educators hold differing views about how difficult it is for students to understand complex equity‐related topics (Valderama‐Wallace and Apesoa‐Varano 2019). Little is known about how students perceive any health equity knowledge gained during their nursing education, nor how student perspectives about their learning align or conflict with explicit curricular content on health equity (Graefe et al. 2024).

## 2 Present Study

To address these gaps, the aims of this manuscript are to (1) explore nursing students' perceptions about using any health equity knowledge gained in school in their future nursing practice and (2) evaluate whether explicit health equity concepts within nursing course documents align with student perspectives of health equity learning in their programs.

After an extensive literature review, two frameworks that were identified as useful to understand this topic are the Commission on Social Determinants of Health (CSDH) conceptual framework (Solar and Irwin 2010) and critical pedagogy (Freire 1970). The CSDH framework describes many levels of influence on health (Solar and Irwin 2010), including the influences of history and historic context, governance and policy and cultural and social values which were identified as areas requiring additional research within nursing education in a previous literature review (Graefe et al. 2024). The CSDH framework is useful for examining health equity content in nursing curricula because the framework: (1) offers a detailed overview of many factors influencing health equity and (2) it considers the healthcare system an intermediary factor that can, to a certain extent, mediate the inequitable impact of the aforementioned influences on health (Solar and Irwin 2010). Nurses, as the largest sector of the healthcare workforce, are an important part of health equity. Critical pedagogy is a philosophy to potentially inform student learning about complex topics related to social injustices and health equity (Freire 1970; Jemal 2017). Critical pedagogy frames education through a lens of critical theory, with knowledge viewed as a social construction shaped by power systems (Freire 1970; Jemal 2017). In critical pedagogy, the goal is for learners to be part of systems change against oppression (Jemal 2017). The learning process involves critical consciousness and conscientisation (see Table 1), including using critical dialogue and reflection to develop awareness of social oppressions and engaging in action for change (Jemal 2017). Critical consciousness has been recently explored among nurses to understand motivation to enact social change (Pirsch et al. 2023). Critical pedagogy was determined to be useful for this study on student learning and application as it includes aspects of critical awareness, reflection and action.

## 3 Methods

The study used a mixed methods explanatory sequential design to quantitatively examine health equity content in curricular documents (i.e., course syllabi, course descriptions, course schedules and nursing program home websites), then qualitatively examine student perspectives regarding health equity content in their curriculum using focus group interviews with students (Creswell and Plano Clark 2017). The document review was intended to investigate the explicit frequency and breadth of health equity topics included in curricula, and the focus group interviews were intended to explore nursing student perspectives regarding program content and application in practice and provide context for the quantitative document findings. According to a recent review of the literature in this area, few studies had evaluated health equity content in nursing programs using multiple methods or mixed methods (Graefe et al. 2024). No studies had used this approach of comparing and integrating curricular content with student perspectives to evaluate the extent of health equity content throughout nursing programs through both perspectives (Graefe et al. 2024). The integration of the two data sources allows for a deeper understanding of what is occurring in programs than either source could offer alone. Results from each data source were analysed separately then integrated and interpreted. The focus group interview guides were created based on findings from the quantitative analysis of the curricula. The mixed methods design allowed for explanation, exploration and contextualisation of the quantitative document findings through the qualitative focus group interviews (Creswell and Plano Clark 2017). This study is reported in line with Journal Article Reporting Standards‐Mixed Methods Article Reporting Standards guidelines (American Psychological Association 2020).

### 3.1 Quantitative Phase

#### 3.1.1 Sample and Recruitment

All accredited prelicensure baccalaureate nursing programs in one midwestern state listed on the state Board of Nursing website were eligible to participate (*n* = 21). The study was promoted during a virtual statewide meeting of nursing programs followed by a recruitment email sent to a listserv of all prelicensure nursing program directors, then individual emails to eligible program directors extracted from the state board of nursing website. An interest and eligibility survey was completed by interested program directors or a designee on REDCap electronic data capture tools (Harris et al. 2009, 2019). Inclusion criteria were prelicensure baccalaureate nursing programs in the state of interest. Exclusion criteria included programs that have made major curricular change in the past year and non‐accredited programs. Programs with major curricular change in the past year were excluded so that the curriculum that was evaluated matched the courses and content that the final semester students in the study had experienced.

#### 3.1.2 Data Collection and Analysis

After meeting eligibility criteria and providing consent, a designee completed a program demographics and characteristics survey via REDCap (Harris et al. 2009, 2019). A designee also provided access to or securely uploaded program documents (i.e., course syllabi and course descriptions/outlines) to Box Secure Storage. Course descriptions and program website content were publicly available and accessed by the first author to download and store as documents in Box. All documents were de‐identified and imported into MAXQDA Analytics Pro 24. Using a directed content analysis approach (Creswell and Plano Clark 2017), the documents were coded for health equity content based on a priori codes derived from the CSDH framework (Solar and Irwin 2010) and exemplars from a previous literature review (Graefe et al. 2024) under three primary categories: cultural and social values, governance and policy and history and historic context (Table 2). These three overarching categories were chosen for this study due to their relevance to health equity according to the CSDH framework (Solar and Irwin 2010) and either: (1) their lack of previous research in nursing education or (2) existing research that identified the categories as gaps (Graefe et al. 2024). Topics that were related to one of the health equity categories of interest were assigned to one or more relevant codes. If the course topic was potentially relevant but vague, it was assigned a broad code (e.g., a standalone reference to ‘Culture’ in a syllabus with no additional context would be coded as *nonspecific cultural references*; see Table 2). New conceptually relevant topics within the overarching categories were incorporated as additional codes. Coded segments were quantitised (Andrew and Halcomb 2009) within MAXQDA and used to report simple descriptive statistics of health equity content within each program and across programs using Microsoft Excel (version 16.94) and RStudio (version 2022.12.0+353). A full list of the subcodes and their overarching content areas from the CSDH framework are presented in Table 2.

**TABLE 2:** Overarching health equity content areas and associated subcodes.

Overarching health equity category	Subcodes
Cultural and social values	Nursing culture Stigma, stereotypes, biases Specific social contexts Social/structural contexts/processes Social position or individual/interpersonal Nonspecific cultural references Nonspecific social references
Governance and policy	Structure/role of government Social participation/civic engagement Policy development Health policies Social policies, broadly Legal and political factors, broadly
History and historic context	Colonisation Racism Nursing history, broadly Historic context of health problems History and historic context, broadly

### 3.2 Qualitative Phase

#### 3.2.1 Sample and Recruitment

The qualitative phase included a convenience sample of nursing students. After the document analysis phase, each program distributed a recruitment email to their final semester students to participate in focus group interviews. Interested students completed an eligibility survey using REDCap. Eligibility criteria were students from the participating prelicensure baccalaureate programs who were nursing majors in their final semester and expected to graduate within the next 6 months. Exclusion criteria were students who transferred into the program and did not complete most of their education requirements at the target institution. Eligible students were contacted by email to schedule a focus group interview time.

#### 3.2.2 Data Collection and Analysis

This portion of the study was guided by a qualitative descriptive approach using a semi‐structured focus group interview guide with open‐ended questions initially based on findings from each program's document review. Critical pedagogy concepts related to critical consciousness informed follow up and probing questions to explore whether and how learners move between learning about equity topics and engaging in action in practice. The first author moderated the focus groups to guide effective group interaction without excessive intervention or expression of biases or assumptions (Holloway and Galvin 2017). The focus group interviews lasted between 44 and 66 min and were recorded and transcribed online through Zoom Workplace (version 6.2.11). The moderator explored potential negative cases and alternative explanations by eliciting various participants' responses and asking whether they had a different perspective (Holloway and Galvin 2017).

Qualitative interview transcripts were imported into MAXQDA. Transcripts were reviewed alongside the recordings and edited for accuracy, then analysed using directed content analysis (Hsieh and Shannon 2005). Initial codes based on health equity topics from the document review codebook and critical pedagogy were used to code the transcripts, with additional codes being added as new themes were identified (Hsieh and Shannon 2005). The analysis process was repeated for each focus group interview, and interviews were compared to determine the major themes (Holloway and Galvin 2017). An audit trail was maintained for trustworthiness, and field notes immediately after interviews and memos of analysis decisions were maintained to minimise bias (Holloway and Galvin 2017; Orne and Bell 2015). The authors of this manuscript represent multiple races, social locations and roles within healthcare and academia that inform our individual experiences and worldviews. The first author attempted to bracket her positionality and beliefs while analysing and interpreting the interviews.

### 3.3 Integration

After document review and focus group interview analysis were completed separately, the results were integrated to explore how the student perspectives contextualise document review findings, and to compare whether there was congruence or conflict between findings. Frequency and breadth of content based on the document review were compared to students' perceived inclusion of content, as well as how students perceived the content to be applicable and integrated into their practice as developing nurses. Integrated findings are presented with a joint display (Table 3) to visualise where data support one another or where there was conflict.

**TABLE 3:** Joint display comparing quantitative and qualitative study findings.

Topic	Courses with > 3 explicit equity content occurrences, *N* = 60	Student focus group interview quotes	Align‐ment^a^
Any health equity content	65.0% (*n* = 39)	Coming into college, I feel like I didn't really have much like knowledge or education about the topic [health equity], so it's very new for me. And so, going from kind of 0 to 100, I feel like now I feel so knowledgeable about it, but also reflecting upon it, there's so much more that I can learn about it. (Group 2, Student 207)	=
		I think, even though some of the topics weren't taught enough, I think we kind of got our toes dipped in enough to kind of open up the spectrum and like, kind of turn the light on inside of our brain. Like, this is what's happening. This is what needs to change. This is how you can help. (Group 1, Student 206)	
Cultural and social values content	56.7% (*n* = 34)	I think that *one* simulation was helpful, but there's obviously more to that than just one type of gender affirming surgery, and there's more than one type of LGBTQ person. So I think it would have been helpful to have, like, honestly, maybe a whole class like going over that stuff. (Group 1, Student 206)	/
		I think our public health course and our public health clinical, and even our leadership class, we kind of looked at … we looked at different topics within healthcare that could be improved upon, and some of the groups discussed barriers to health equity like financial barriers and insurance and different things like that. (Group 1, Student 204)	
		One big cultural aspect that I've seen a lot in clinical is just the use of a translator. And I feel like the hospital systems could definitely do a better job, just with that whole system. Cause it's definitely … it's not the best way they could go about it. It's on an iPad. The doctor talks to the translator, the translator talks to the patients, and I feel like that definitely makes the healthcare interaction more difficult because of like patients and families having a difficult time understanding and getting the questions that they have across. So I feel like that's definitely a cultural aspect that I see a lot on a day to day basis that could be improved. (Group 2, Student 207)	
		I had an experience in my paediatric rotation where there was this child … It really impacted me to see this family that had fled from [country redacted], and this mother had [multiple children], and her son was unable to walk on his own. He was developmentally impacted. He had a lot of complex healthcare or health needs. And seeing how the hospital addressed the family and the resources that were available to them kind of surprised me, but I was thankful for it, to see the support that this immigrant family received. But it definitely made me think about the system, and how many barriers to being there to support her son there were. Like she had to get a taxi every time she wanted to come to the hospital. She had other kids that she was enrolling in the school systems, and so many of these, like socioeconomic factors came into play as the hospital was supporting this family. (Group 1, Student 204)	
Governance and policy content	23.3% (*n* = 14)	We did have to deal with some legal issues at clinical. Like you had patients who came in who didn't have insurance. And so then that's where, you know, the government and insurance policies and all of that come into play. But I do really feel like that was something that was kind of skipped over a little bit more so in classroom settings. (Group 1, Student 205)	=
		I feel like the only sort of like government information we got was like, maybe in ethics class. We learned a teensy weensy bit about just different government stuff, insurance, Medicare, Medicaid. I think it'd be really helpful if we had kind of like a ‘current policies and laws’ class, especially with so much changing in health law and policies in recent years. (Group 1, Student 206)	
History and historic context content	5.0% (*n* = 3)	I agree [with previous student statement]. Nothing really comes to mind in terms of the history [related to health equity]. I feel like we've, throughout school, whether it was in the classroom, or just in our personal lives, we've kind of like picked up on things. But as far as emphasis in the curriculum on the history, I think it's not really a focus. And if we think about like social history, like a lot of curricula across different subjects, I think it has been whitewashed. Like we don't talk about the origins of—I mean, [other student] mentioned, we've heard of studies and things [referring to Tuskegee Syphilis Study]—but [not] the focus on the historical landscape that has evolved into what we have now. (Group 1, Student 204)	=
		I think the first time I had ever even heard the word colonisation in a class was this spring … And it was like, just grazed over … I mean, I feel like we talked about racism a lot generally, but not like the historical context of it. And we all know that people of colour face health inequities. But I don't know if we talked about why—actually, that's not true! But this was specific to my public health clinical. My clinical instructor was really great, and we did a long discussion about, like, the transition from slavery to incarceration, and how it's just like one big like continuum of slavery, basically, but super disproportionately affects people of colour. But other than that, like in our big classes, I don't really remember that much. (Group 2, Student 203)	
Any equity content in clinical (*N* = 15) vs. non‐clinical (*N* = 33) courses^b^	Clinical: *n* = 8 (53.3%) Non‐clinical: *n* = 20 (60.6%)	There's so many parts of nursing that you have to just experience. There's only so much you can learn in a classroom. And I feel like even if there's some areas that were lacking in our curriculum as far as these health equity topics, I think [nursing program name] does a good job of placing us in situations where we can see them in the real world and learn to work through them as we face them. (Group 1, Student 204)	≠
		I feel like I got the most insight into that at a clinical setting, and not so much in a classroom setting just because we did have to deal with some legal issues at clinical … But I do really feel like that was something that was kind of skipped over a little bit more so in classroom settings. (Group 1, Student 205)	

*Note:* Qualitative findings from students are compared to overall quantitative findings across programs to contextualise the quantitative findings. Three documents (course syllabus, description, and outline/schedule) were analysed for each course. Courses with more than three equity content occurrences across their documents are reported in this table to capture courses with potentially greater frequency or depth of content.

^a^ = indicates general alignment between quantitative and qualitative findings. / indicates a mix between alignment and conflict between quantitative and qualitative findings. ≠ indicates conflict between quantitative and qualitative findings.

^b^ One program included a clinical/lab/simulation component with all of their courses and thus was excluded from this comparison.

### 3.4 Ethical Considerations

This study was submitted to the University of Minnesota Institutional Review Board (IRB#: STUDY00020765) and determined to be exempt from review on 11/27/23. All participants received detailed written information about the study. Participants were informed that participation was voluntary and they could withdraw at any time. Collected data were de‐identified and stored separately from potential identifiers within Box. Confidentiality was maintained throughout the study.

## 4 Results

### 4.1 Quantitative

Three baccalaureate nursing programs participated in the study, including public and private institutions with a variety of institution sizes (i.e., ranging from 1000–2999 to > 10,000 full‐time equivalent students) and designations (e.g., faith‐based, land grant). All were 4‐year programs, with most nursing courses occurring in the final 2 years. A total of 197 documents from 60 total nursing courses from the programs were analysed (see Table 4). An overview of the percentage of courses with equity content across the programs, broken down by overarching equity topic, is presented in Table 3. Of all courses, 88.3% (*n* = 53) included at least one occurrence of health equity content across their syllabus, course description and/or course schedule/outline and 65.0% (*n* = 39) of courses contained more than three total equity content occurrences. There were a mean of 11.0 health equity content occurrences per course. Although findings varied between programs, each program shared similar overall findings, with each having the most content regarding cultural/social values and the least content on history/historic context. There was no explicit content in any documents from any program on whiteness/white privilege, historic racism or colonisation/colonialism. Further descriptive findings from the quantitative arm of the study are beyond the scope of this manuscript and will be reported elsewhere.

**TABLE 4:** Type and number of documents analysed by nursing program.

Document type	Number of documents		
	Program 1	Program 2	Program 3
Syllabus	40	28	12
Course description	19	28	12
Course schedule/outline	19	28	11
Total documents (*N* = 197)	78	84	35

### 4.2 Qualitative

Two focus group interviews were conducted with six students from one of the participating programs. Three students from a second program completed the eligibility survey but did not respond for focus group interview scheduling. Students' mean age was 21.8, and all identified as White women from the Midwest. There was some variation in sexual orientation, spiritual belief system and rurality of the area where they primarily grew up. In addition to the primary health equity topic areas studied, themes from the focus group interviews included dimensions of critical consciousness, experiential learning and learning through the unknown.

#### 4.2.1 Cultural and Social Values

Student experiences relating to the depth and frequency of cultural and social values content are included in Table 3. Students described many examples of various social determinants of health impacts on patients, of assessing patient cultural values and meeting cultural needs.

I think I learned so much about through clinical rotations in terms of like, you need to use an interpreter if the patient can't understand you. That's just how it should be. They need to be able to communicate their needs and preferences with you … Or just knowing that a patient doesn't have access to food and then helping them get set up with like SNAP [Supplemental Nutrition Assistance Program] and WIC [Women, Infants and Children Nutrition Program] or something like that. (Group 2, Student 203)

Students mentioned that they were taught about racism within their curriculum. One student shared an experience of witnessing racism against a patient who was incarcerated, describing:

Some of the nurses were super great about being like, ‘it's just any other patient, you have to respect their wishes, kind of put aside whatever their social situation is, and kind of just treat the person versus treat like their history’. And some of the nurses were pretty clearly against providing care for him, and he happened to be an African American man, and I even noticed one nurse was a little bit—Not a little bit—She was pretty blatantly racist, which was unfortunate. And I don't know if maybe she would have felt comfortable to say what she said had he been a non‐incarcerated Black person versus an incarcerated Black person. I think that actually made her feel more empowered to make those racist comments. But I don't know for sure. (Group 2, Student 203)

Students described important learning experiences that arose when working with LGBTQIATS+ patients in clinical and simulation settings.

We had a simulation taking care of a post‐operative gender affirming surgical patient … That was something I've never experienced before, and just the way that we prepared for it was really eye opening and even the patient themselves. It was nice to get that experience before being thrown into it working as a nurse. (Group 1, Student 206)

#### 4.2.2 History and Historic Context

Nursing students perceived that very little history and historic context content was presented to them in their program (see also Table 3).

We touched a little bit on some of the history stuff … We learned of these horrible medical studies that happened back in the day, like the Tuskegee Syphilis Study and things along that line we learned a tiny bit about … But it wasn't really much. It was just a sprinkle. (Group 1, Student 206)

When discussing colonialism and the history of systemic racism, a student in one focus group interview said that learning about colonialism and historic racism was or would be important to their development as a nurse.

Personally, I would really appreciate having that background knowledge [about historic context]. I feel like I'm kind of lucky because I did get a good amount of education about kind of the history of it, generally—not related to healthcare, but I can apply it to healthcare—like through high school and whatever. But to kind of maybe have a bit of a discussion about how we got to where we are would maybe clear up why things are the way that they are, and kind of backtrack into how we can fix them. (Group 2, Student, 203)

Another student in the group was more unsure of how to fit historic racism and colonialism content into the curriculum despite thinking it would be helpful.

I feel like looking back, maybe we could say, ‘yeah, that'd be helpful’. But at the same time, we have so much content that we're learning on a day‐to‐day basis. I feel like [it's] one of those things would be another topic just shoved into our leadership class that would be briefly touched over. But I definitely think it'd be helpful to learn about, but also challenging to fit in a spot for it. (Group 2, Student 207)

Students also discussed how learning about historic roots of medical mistrust were important in understanding current patient attitudes and perspectives.

I just think about the Tuskegee syphilis trials; it's like—I would be terrified to seek any sort of medical treatment if I knew that was something that was even in the realm of possibilities. And like, it happened not that long ago. So, it's just kind of understanding maybe why people hesitate to seek medical care from a historical perspective, not even lack of access, just like fear and knowledge that they'll face discrimination. (Group 2, Student 203)

Also, as [Student 206] said, [we learned] about the mistrust in the healthcare system by certain populations, and why that is, and how we can meet patients where they are at with their mistrust, and how we can understand where that came from. (Group 1, Student 208)

#### 4.2.3 Governance and Policy

Student perceptions of the amount and depth of content related to governance and policy in the curriculum were mixed (see Table 3). Students were able to discuss the role of some government programs like WIC, Medicare and Medicaid; however, students wanted more information about these programs, other programs available to patients, and how they can actually go about creating policy change as nurses.

Reflecting on it, I'm kind of like, oh yeah, I guess we didn't really talk about that, because I feel like the extent of what we get on the government is: ‘[for] the supports out there, there's all these stipulations, so they don't work for everybody. There's a lot of people that slip through the cracks’. And we're just kind of told, ‘it's important that nurses *advocate*, for *everyone*’. It's like, okay? And so, I think the extent of my understanding of the role the government plays is: Medicare part A, Medicare part B, and that's about it. (Group 1, Student 204)

Okay, I would agree with [Student 205] that we really didn't go into the government's role into health. Only kind of touched on it a little bit in our leadership course … But that was more from like an environmental aspect and global climate change … I'm a public health minor, so I learned most about what I know from those classes as opposed to in nursing, which kind of seems a little backwards, and I really wish I would have learned more about kind of how we, as nurses, can best support policy changes, and especially in advocating for healthcare for all. (Group 1, Student 208)

When describing witnessing cycles of patients experiencing homelessness coming into an inpatient mental health unit and then being discharged back into homelessness, one student went on to describe the connection to governance and policy, stating:

A lot of the patients were telling me about how their window—I was at [a hospital where] the window overlooked [an event center], and a lot of them had a lot of hatred towards the government, because a couple of years ago, when they got the new [event center], they were able to watch this, like, multi‐million‐dollar [event center] being built next to the unit, and they felt so angry because they feel that they have no support or no resources from the government to help them with their cycle [of homelessness] or their mental health. And then they see this million‐dollar [event center] being built. (Group 2, Student 207)

#### 4.2.4 Critical Consciousness

As students discussed examples of what they felt they had learned about health equity and how they found it relevant and applicable to their practice as soon‐to‐be nurses, they demonstrated components of critical consciousness (see Table 5). Students described times that they became aware of inequities and limited supports through clinical and simulation experiences and classroom dialogue (Table 5). Students also described that they each came into the nursing program with different levels of equity‐related knowledge and understandings of privilege. Student 203 in Group 1 stated, ‘I think some people come in with a lot of knowledge, and some people come in with none’.

**TABLE 5:** Focus group themes: Student application of learning about health equity.

Student quote	Dimension of theme reflected in quote
Conscientisation, critical consciousness (CC) and praxis	
I know there was a few people in my cohort in [location redacted] that every time we would briefly mention some of these [health equity] topics, they would seriously ask, ‘Why is this the only time we're learning about it?’ And that was really eye opening, like, ‘yeah, why *is* this the only time we're learning about it?’—Group 1, Student 206	Developing CC through critical dialogue and critical reflection
I feel like my mental health clinical, was my most eye‐opening experience … Some of the patients I saw on my mental health rotation, it was like their third or fourth time on this unit, because once they get good on the unit, they get their treatment, they get into a good mental situation, then they're released back, and then it's like they just get bad again because they're put back into homelessness. And it was definitely eye opening for me to see that just because I've never been exposed to that kind of situation, and I grew up in the suburbs of [city redacted], and I wasn't around a lot of homelessness. And so I feel like it almost changed my attitude toward it and made me a more empathetic person.—Group 2, Student 207	Developing CC through critical reflection
I would say, I grew up in a very small town less with than a thousand people and I was not aware of literally none of this growing up. I think, coming to nursing school and going through this program and seeing the things that I've seen at clinical … It's astonishing the things that I wasn't aware of. I think, being aware of it now, will definitely make me a better nurse, just like [name redacted] and [name redacted] and [name redacted] said.—Group 1, Student 206	Developing CC through critical reflection
Kind of like [Student 207] said, I went to a very diverse high school, but I lived a really privileged life, like on its own, and so while I *kind of* saw the inequities that were happening, it just isn't the same as like being able to experience it first hand and provide different types of care.—Group 2, Student 203	Developing CC through action as a nursing student
I would say that through all of my time in clinicals, and actually experiencing the state of healthcare today has truly opened up my eyes to the need for change in our country. And I guess it wasn't something I ever thought about, the same as [name redacted] and [name redacted]. I never thought twice about going to the doctor for sake of not being able to pay because of how privileged I've been raised. And I guess nursing and being in those clinical places even for the short time … it's instilled this drive to want to change things and want to inspire those in power to change things, because not everyone gets to see this—so many different people's perspectives and different situations. And a lot of people don't understand why—they don't believe in a more universal health care approach because they've only seen their own situation. They don't understand others' [situations], and being clinical has definitely showed me that we need change and reform in our system.—Group 1, Student 208	Engagement in critical reflection resulting in the desire to create change
I think all the things I've seen have enhanced my compassion and empathy, and desire to be a presence in the healthcare field to advocate for the people that we see.—Group 1, Student 204	Desire to create change/action
I can kind of think of a patient I had that identified in the LGBTQ community. They were younger and they were sort of hiding it from their parents … It was a little bit hard to navigate at first, especially because you're like, well they prefer to be called this, I want to call them this, and they know that this is what they want to be called. But you don't want to create any conflict, or like ‘out them’ when they don't feel comfortable doing that in front of their parents. So that was kind of difficult for me … But I'm glad I had that experience, and I definitely know what I can do better, what I can change, or what I can keep doing the same for the next patient I have.—Group 1, Student 206	Critical reflection to inform future action
We have to educate other people, and we have to not just rely on ourselves but also rely on the support of others. And like, spread this knowledge and this information, and spread what we see, and *describe* these patient scenarios that we find, and really hope that it can, like, flip a switch. And just other everyday people who aren't in the hospitals … just because it really starts outside of these hospitals and these facilities. Right? I mean, we are the ones that see it and treat it. But I mean, it really starts outside. And I feel like outside of the hospital, everyone plays a role. And I think while I guess *I* feel confident that I have been given the right tools, so many other people haven't, and so I feel like it's not up to me single handedly. But I guess I play a role in educating other people as well. So, that's kind of really where the change is going to happen.—Group 1, Student 205	Critical reflection and motivation to create change and engage in action
One specific clinical experience would be one of the moms that I had taken care of during my public health clinical … She was pregnant, and she was a teenager, undocumented, and living in her mom's house, but it was kind of unstable … and the public health nurse that I was working with did a lot of like care coordination to try to find, ‘Okay, here's what shelter you need to go to’, and those sorts of things. And it was a lot of trying to figure out what little scraps of help that you could get for a patient and a mom who was just completely struggling, and on her own in the world. And it was really powerful, but also extremely sad to see how a lot of people are just left to, I don't know, without any sort of help or resources, and being so scared and terrified, and being pregnant on top of all of that … Hearing her story and hearing how my nurse kind of coordinated those things for her and with her was powerful.—Group 1, Student 208	Witnessing a preceptor engage in action
Learning through the unknown	
I was in the inpatient mental health unit for paediatrics, for kids. And it was kind of similar to what [another student] shared. But the patient identified in the LGBTQ+ population, as being like, using different pronouns than what maybe their family had called them all their life … And it was really hard trying to have family therapy and conversations with their parents … And navigating that, I relied a lot on my nurse preceptor for, because I didn't really know … It's really tricky waters to navigate, and I don't think we learned anything about that in school. So I was kind of going based off what my nurse was doing, and she did a wonderful job of kind of balancing that.—Group 1, Student 208	Learning to navigate a challenging scenario by following guidance of a preceptor

*Note:* The critical consciousness‐related themes within this table are informed by Freire (1970) and Jemal (2017) (see Table 1). The fourth theme of learning through the unknown was an additional theme identified during analysis. Examples of evidence come from student focus group interviews.

Student moments of learning and becoming aware of social realities and injustices were also connected to some examples of student commitment to action. One student, after reflecting on her privilege growing up and having learned about social realities that many patients face, said, ‘I feel like it makes me a better nurse now, seeing this in action. Just because, it makes me want to work harder to find a solution for these patients, a more long term, lasting solution’ (Group 1, Student 205). At the same time, some students acknowledged understanding how to make change on an individual level but were not sure about how to go about creating systemic change.

Big picture, I think I *personally* understand how in my *individual* actions and interactions with patients, I can kind of support their health equity outcomes in terms of providing the resources that I'm aware of and connecting them with other people who are more knowledgeable than me. But I *don't* think we learned enough about how to make *systemic* changes. Because I could do everything in my power to help my one patient overcome some of the barriers, but at the end of the day, that's one patient. Like, that's not going to fix the whole big problem that is health and equity. (Group 2, Student 203)

There was also some evidence of cyclical reflection and action by students, or student observation of action by their mentors, and many students acknowledged their need for ongoing learning (Table 5). Ultimately, these examples showed initial evidence of components of conscientisation, critical consciousness and praxis but focused more on reflection than action at this time, as many of the scenarios resulted in students *wanting* to engage in action in the future but not having yet done so and feeling unprepared to do so.

#### 4.2.5 Experiential Learning

Students in both focus group interviews emphasised the impact of simulation and clinical experiences on their learning about health equity (Table 5).

But I think that one of my bigger takeaways from learning about this [health equity] in my program was definitely, I would like to really reiterate, the simulations were super powerful, and also our clinical experience in public health. (Group 1, Student 208)

Students also particularly noted the impact of seeing firsthand how equity‐related topics affect their practice and their patients in clinical settings. For example, one student described learning about the use of interpreters and screening for SDOH like food insecurity and said, ‘Until you put your hands on it and kind of like see how complex of an issue it [SDOH] is, I feel like you can't really grasp how significant it is in people's health outcomes’ (Group 2, Student 203).

#### 4.2.6 Learning Through the Unknown

Another theme that emerged from the focus group interviews was the experience of learning through the unknown (Table 5). Students described how navigating equity‐related scenarios can be complex and difficult. One student described a challenging scenario when working with a family who was undocumented immigrants.

And then getting emergency health insurance and emergency assistance to cover medication costs, like the costs were thousands of dollars for these medications that the patient seriously needed to survive and live day to day, and they really had no way to pay for this stuff … Every time they get sick they have to be admitted to the pediatric ICU, and it's just costs and costs and costs, stacking up on each other, and being illegal immigrants, you don't really have many options, like, they can't really get health insurance here. So, navigating all that was really hard and kind of confusing for me, because I wasn't super familiar with all that stuff. (Group 1, Student 206)

Students also discussed having to navigate difficult conversations and scenarios, and how they were grateful to practice this either in a simulation setting or with the help of or by observing a preceptor in the clinical setting on how to navigate these situations.

There is one simulation where there was the son and the dad, and I think the son had a gay partner, and the dad was not approving of it. And so, in the simulation we kind of had to like navigate that conversation with the family, which is definitely useful to have, because we'll run into that in clinical practice. (Group 2, Student 207)

### 4.3 Integration

Integrated findings comparing student perspectives to overall document findings across all programs are presented in a joint display in Table 3. In some areas, student perceptions aligned with document analysis findings, while in other areas the findings diverged. There was the most equity‐related content identified in program documents across all programs related to cultural and social values. This generally aligned with student perspectives, as students provided many examples of various social determinants of health impacts on patients and of assessing patient cultural values and meeting cultural needs. A divergence was found between content related to the care of LGBTQIATS+ patients, namely that there was very little curricular content in this area, but students described important learning opportunities that they encountered in clinical and simulation settings. There was very little content on history and historical context related to health equity across curricular documents, nor did students perceive that much of the content was presented to them. Program documents contained some content on governance and policy, which was similar to student perceptions. Of the programs that did not have a clinical component in every course, their courses without clinical/simulation/lab had more equity content in curricular documents than their courses with a clinical/simulation/lab component. This finding diverged from the perceptions of students in one of those programs, as students emphasised the impact of seeing firsthand how equity‐related topics impact their practice and their patients in clinical settings. In some cases, students described learning about equity‐related content more in their clinical and simulation experiences than in the classroom setting.

## 5 Discussion

This article reported findings from a mixed methods study, including quantitative document analysis of explicit health equity content across three nursing programs, qualitative student focus groups regarding student perceptions of content and their application of any learning, and integration of the two sources of data. Overall, explicit health equity content varied across courses and between health equity content areas, with the most content related to cultural and social values and the least content related to history and historic context. There was more equity content in courses that did not include a clinical/simulation/lab component than in courses that did. Students described experiences learning about equity content in courses and experiential learning settings. Students described some content areas of strength (social determinants of health, cultural factors) and other areas that were lacking (policy, history). When describing their application of learning in practice settings, students demonstrated aspects of critical consciousness, particularly becoming aware of injustices, engaging in critical reflection and having motivation to create change. Generally, qualitative and quantitative findings aligned across content areas; however, students described that clinical experiences were meaningful to their learning about health equity, and sometimes more so than classroom experiences, which conflicted with the amount of explicit content in course documents.

The finding that history and policy topics were lacking compared to other topics and that students described critical consciousness components of critical awareness, reflection and motivation with less action when it came to equity topics aligns with current literature on the need for nursing activism (Chinn and Kennedy 2023). Some students recognised the need for activism on behalf of their patients, but they had less understanding of how to create systemic or policy change. Students also expressed a need for more specific and concrete examples of nursing involvement in policy, such as those described by Kuehnert et al. (2022). Student learning and competence in these areas might be strengthened in nursing programs given a number of subcompetencies focused on such topics in the American Association of Colleges of Nursing (2021) *Essentials*. Nurse educators can consider linking together history and policy content to provide historic and current examples of health care workers' action toward equity, such as those described by Weitzel et al. (2020), Dawes (2025) and Chinn and Kennedy (2023), to help students make the connection between course content and activism as nurses.

Some differences in findings of equity content between data sources, such as the difference between clinical and non‐clinical courses that was perceived inversely by students as compared to curricular documents, highlight the limitations of document review as an exclusive source of curricular evaluation and decision making. When evaluating or redesigning curricula, programs should consider efforts beyond content mapping and use multiple data sources to provide a holistic understanding of their curricular content. Students' perceived benefits of their experiential learning align with the literature highlighting the importance of quality clinical preceptors and clinical placement experiences (Rosli et al. 2022). Additionally, the difference between student perspectives and curricular documents in this case may reflect differing clinical opportunities that students experience within a course based on individual clinical placements, clinical preceptors and clinical nursing faculty. Nursing programs should consider reviewing whether their clinical experiences are providing opportunities for students to engage in equity‐related learning and how they can better reflect such learning in curricular documents.

This study has a number of strengths. First, the mixed‐methods design is unique among studies on health equity content in nursing curricula. This is the first study, to our knowledge, that compares explicit curricular document findings to student perspectives and perceived application of learning in this area (Graefe et al. 2024). Additionally, the inclusion of multiple nursing programs in the document analysis is a strength as most research in this area has either focused on individual programs or has lacked depth and detailed analysis when including multiple programs (Graefe et al. 2024).

This study also has a few limitations. Despite including multiple programs, the program sample is limited geographically to the US Midwest. This limits the generalisability of the findings as there may be cultural considerations or possibly programmatic considerations that are regionally based. The student sample also reflects the predominance of White women nurses in the US (Smiley et al. 2023). Understanding how this sample of White women perceived equity content and application is important, but future studies should explore the perceptions of learning about health equity among students of colour and American Indians, additional gender identities, from different nursing programs, and even in other countries. While nursing in the US is still a profession that is dominated by White women, this is changing rapidly with the profession becoming more representative of the communities served (National Academies of Sciences, Engineering, and Medicine 2021). This is a welcome change as it enables the nursing profession to be more attuned to the realities and healthcare needs of various communities in the US. The cross‐sectional design of this study also only represents a snapshot of curriculum and student perspectives and does not allow for any causal inferences (Hulley et al. 2013).

## 6 Conclusion

In conclusion, this mixed methods study provides rich insight into students' perceived learning and application of learned health equity‐related content and areas of alignment and conflict between student perspectives and explicit content in curricular documents. Students perceived learning about many health equity topics, especially cultural and social values, but identified key content gaps within history and historic context and policy‐related topics. Students also described processes of conscientisation but had not yet engaged in much actual praxis with critical action. The integration of curricular document findings with student perceptions also provided a better holistic understanding of what learning opportunities exist within curricula, such as the importance of experiential learning, as well as potential areas for strengthening curriculum and for future research. The findings from this study can be used for nursing programs as they explore curricular redesign, integration of equity content and/or development of critical consciousness among their students. For example, programs can assess the content gaps noted in this study within their own programs and evaluate existing or new opportunities for student application of health equity learning in their program. Researchers should consider further exploring the sufficiency of health equity content and student learning in nursing education within additional contexts and samples.

## Author Contributions

All authors have agreed on the final version and meet at least one of the following criteria (recommended by the ICMJE*): (1) substantial contributions to conception and design, acquisition of data or analysis and interpretation of data; (2) drafting the article or revising it critically for important intellectual content. *http://www.icmje.org/recommendations/.

## Conflicts of Interest

The authors declare no conflicts of interest.

## Data Availability

The authors have nothing to report.
